# Association of *FCGR2A *and *FCGR2A-FCGR3A *haplotypes with susceptibility to giant cell arteritis

**DOI:** 10.1186/ar1996

**Published:** 2006-07-17

**Authors:** Ann W Morgan, Jim I Robinson, Jennifer H Barrett, Javier Martin, Amy Walker, Sarah J Babbage, William ER Ollier, Miguel A Gonzalez-Gay, John D Isaacs

**Affiliations:** 1 Leeds Institute for Molecular Medicine, University of Leeds, Leeds, UK; 2 Instituto de Parasitología y Biomedicina López Neyra, CSIC, Granada, Spain; 3 The Centre for Integrated Genomic Medical Research, The University of Manchester, Manchester, UK; 4 Rheumatology Division, Hospital Xeral-Calde, Lugo, Spain; 5 School of Clinical Medical Sciences (Rheumatology), University of Newcastle-Upon-Tyne, UK

## Abstract

The Fc gamma receptors have been shown to play important roles in the initiation and regulation of many immunological and inflammatory processes and to amplify and refine the immune response to an infection. We have investigated the hypothesis that polymorphism within the *FCGR *genetic locus is associated with giant cell arteritis (GCA). Biallelic polymorphisms in *FCGR2A*, *FCGR3A*, *FCGR3B *and *FCGR2B *were examined for association with biopsy-proven GCA (*n *= 85) and healthy ethnically matched controls (*n *= 132) in a well-characterised cohort from Lugo, Spain. Haplotype frequencies and linkage disequilibrium (*D*') were estimated across the *FCGR *locus and a model-free analysis performed to determine association with GCA. There was a significant association between *FCGR2A*-131RR homozygosity (odds ratio (OR) 2.10, 95% confidence interval (CI) 1.12 to 3.77, *P *= 0.02, compared with all others) and carriage of *FCGR3A*-158F (OR 3.09, 95% CI 1.10 to 8.64, *P *= 0.03, compared with non-carriers) with susceptibility to GCA. *FCGR *haplotypes were examined to refine the extent of the association. The haplotype showing the strongest association with GCA susceptibility was the *FCGR2A-FCGR3A *131R-158F haplotype (OR 2.84, *P *= 0.01 for homozygotes compared with all others). There was evidence of a multiplicative joint effect between homozygosity for *FCGR2A*-131R and HLA-DRB1*04 positivity, consistent with both of these two genetic factors contributing to the risk of disease. The risk of GCA in HLA-DRB1*04 positive individuals homozygous for the *FCGR2A*-131R allele is increased almost six-fold compared with those with other *FCGR2A *genotypes who are HLA-DRB1*04 negative. We have demonstrated that *FCGR2A *may contribute to the 'susceptibility' of GCA in this Spanish population. The increased association observed with a *FCGR2A-FCGR3A *haplotype suggests the presence of additional genetic polymorphisms in linkage disequilibrium with this haplotype that may contribute to disease susceptibility. These findings may ultimately provide new insights into disease pathogenesis.

## Introduction

Giant cell arteritis (GCA) is a common chronic granulomatous vasculitis that is restricted to the over-50 population and thus serves as a paradigm for ageing-related immunopathology. Permanent ischaemic lesions, predominantly irreversible blindness, occur in 15% of patients due to hyperplasia of the intimal layer of involved arteries and non-thrombotic luminal occlusion. Some patients present acutely with blindness, secondary to anterior ischaemic optic neuropathy or central retinal artery occlusion, whereas others present with a systemic inflammatory syndrome [1]. High-dose steroids are conventionally used to prevent these ischaemic complications, but in an elderly population this leads to a high incidence of adverse events [2].

There is some evidence that GCA is an antigen-driven, autoimmune disease. One of the earliest changes within the vessel wall is the accumulation of dendritic cells within the adventitia, which are believed to initiate and maintain antigen-specific adaptive immune responses, following an as yet unknown vascular insult [3]. The familial clustering of GCA supports a genetic component, and there is a strong association with HLA-DRB1*04 in many different populations [4]. Within cohorts of biopsy-proven GCA, HLA-DRB1*04 is associated with systemic signs and symptoms [5], visual manifestations [6] and corticosteroid resistance [7]. Many other studies have examined genetic variants in key components of immune and inflammatory pathways known to be activated in this disease. Associations with polymorphisms in genes encoding tumour necrosis factor [8], interleukin-4 [9], intracellular adhesion molecule-1 [10], vascular endothelial growth factor [11,12] and endothelial nitric oxide synthase have been reported in some cohorts [13,14], although many are awaiting replication in a second population. Variant alleles of mannose-binding lectin, which result in low levels of mannose-binding lectin protein, are also associated with GCA, perhaps through modulation of phagocytic function [15].

There is some epidemiological evidence, such as clustering of cases in space and time, that infection may act as a trigger for both GCA and polymyalgia rheumatica [16]. Organisms proposed include parvovirus B19 and *Chlamydia pneumoniae*, but the evidence remains inconclusive [17]. Infections may lead to vasculitis through various mechanisms: for example, interactions between microbial ligands and endogenous molecules, impairment of pathogen clearance, molecular mimicry, modification of self epitopes into 'neo-antigens', or failure to down-regulate the alloimmune response [18]. The ageing process itself leads to a functional decline in adaptive and innate immune responses, known as immunosenescence, in association with an increased susceptibility to infections, malignancies and autoimmune/inflammatory disorders. Although GCA-specific autoantibodies have not been described, antibodies against a broad range of human autoantigens have been observed in both types of GCA [19]. Anticardiolipin antibodies have been reported, particularly in biopsy-positive GCA [20], and often disappear with steroid treatment [21]. Furthermore, the pathogenicity of anti-endothelial antibodies, which have been demonstrated in up to 50% of GCA patients, remains to be elucidated, but such antibodies have the potential to mediate Fc gamma receptor (FcγR) cross-linking and trigger downstream effector functions [22].

The FcγRs, which bind IgG containing immune complexes/IgG autoantibodies, have been shown to play important roles in the initiation and regulation of many immunological and inflammatory processes and to amplify and refine the immune response to an infection. Activating FcγRs (FcγRI, FcγRIIa, FcγRIIIa, FcγRIIIb) potentiate phagocytosis in response to IgG-containing immune complexes/opsonised micro-organisms and trigger the oxidative burst, degranulation, maturation and release of cytokines, including tumour necrosis factor-α [23-25]. FcγR-mediated endocytosis by antigen presenting cells, such as dendritic cells, results in efficient MHC class I and II presentation [26]. FcγRIIIa cross-linking additionally stimulates antigen-dependent cellular cytotoxicity by natural killer cells and macrophages. Conversely, FcγRIIb contains an inhibitory motif in the cytoplasmic tail that abrogates cellular activation and down-regulates the antibody response, thus acting as a negative feedback mechanism [23-25]. Polymorphic variants that increase the expression or affinity of these IgG receptors, or enhance their ability to bind specific IgG isotypes, may, therefore, play an important role in determining both the inflammation mediated by IgG (auto)antibodies and IgG-containing immune complexes and/or the susceptibility to specific infections that may be associated with triggering vascular/inflammatory disease.

We have examined the hypothesis that the *FCGR *genetic locus is associated with susceptibility to GCA in a previously well-characterised GCA cohort from north-western Spain.

## Materials and methods

### Giant cell arteritis patients and controls

This was an allelic association study where *FCGR2A*, *FCGR3A*, *FCGR3B *and *FCGR2B *alleles and pairwise haplotypes were examined in a well-characterised GCA cohort from Lugo, Spain [6,27]. Briefly, all patients and controls were of local Spanish descent and originated from the area surrounding Lugo, Galicia, in north-western Spain. They comprised 217 individuals (132 healthy controls and 85 GCA patients). All patients were recruited from Xeral-Calde Hospital (Lugo) and all patients fulfilled the 1990 American College of Rheumatology criteria for the classification of GCA and had a positive temporal artery biopsy [28]. All controls were healthy volunteers, who could trace their ancestry in the region for at least three generations. Ethical approval was obtained from the respective Local Research Ethics Committees.

### *FCGR *genotyping

Genotyping at the *FCGR *locus is complex in view of the high level of structural homology between the three class II (*FCGR2A*, *FCGR2B *and *FCGR2C*) and two class III (*FCGR3A *and *FCGR3B*) receptors. Gene duplication at this locus is believed to have occurred as a result of an unequal crossover event [29] with subsequent divergence of biological functions. The functional *FCGR2A*-131H/R, *FCGR3A*-158F/V and *FCGR3B*-NA1/NA2 polymorphisms and 3' untranslated region *FCGR2B*-1206G/A single nucleotide polymorphism were genotyped using assays that have previously been validated in our laboratory [30,31]. *FCGR2A *genotyping was performed by direct sequencing for >80% of samples. The *FCGR *gene order from centromere to telomere at chromosome 1q23 is *FCGR2A*, *FCGR3A*, *FCGR2C*, *FCGR3B*, *FCGR2B *[31,32].

### Statistical analyses

Statistical analyses were performed using the Stata statistical software (Stata Corporation 2005, Stata Statistical Software: Release 9.0, College Station, Texas, USA) unless otherwise stated. The power calculations for this study were based on the *FCGR *allele frequencies observed in the UK population [31]. A cohort of 85 GCA cases and 130 controls provides 80% power to detect an odds ratio (OR) of 2.5 or 2.9 for a dominant and recessive mode of inheritance, respectively, assuming an allele frequency of 0.35 (5% significance level); for an allele frequency of 0.5 the corresponding ORs are 3.2 and 2.5). Hardy-Weinberg equilibrium was investigated in each control population using an exact test. Allele and genotype frequencies were compared using 2 × 2 and 3 × 2 contingency tables, respectively. Two-sided P values below 0.05 were considered statistically significant throughout.

ORs and their 95% confidence intervals (CI) were calculated to quantify the magnitude of all significant associations, as an approximation of the relative risk. Haplotype frequencies were estimated pairwise across the *FCGR *locus using the Estimating Haplotypes PLUS (EHPLUS) program [33], which also calculates the empirical significance (P value) of overall linkage disequilibrium. The standardised disequilibrium coefficient (*D*') was calculated for each pair of *FCGR*s, utilizing the gene order derived from our electronic contig. The heterogeneity test within the Permutation and Model-free analysis program was used to test for association with disease. A permutation procedure implemented in this program enabled 1,000 permutations to be performed [33].

Association of *FCGR *haplotypes with GCA was investigated further using the haplotype trend regression approach proposed by Zaykin and colleagues [34] for dealing with uncertain phase. In this method, logistic regression can be used to relate disease status to an individual's haplotypes; where these are not known with certainty all haplotypes consistent with the genotypes are included as predictors, weighted by their probabilities (based on the estimated haplotype frequencies). This approach estimates the effect on risk of each haplotype assuming the effect of the two haplotypes is independent. Stepwise regression analyses were also used to investigate a possible interaction with HLA-DRB1*04 [35].

## Results

### Association of *FCGR2A*, *FCGR3A*, *FCGR3B *and *FCGR2B *with giant cell arteritis

There was no evidence of departure from Hardy-Weinberg equilibrium in the control group for any genotyped polymorphism.

There was weak evidence of a difference in the distribution of *FCGR2A*-131H/R alleles (*P *= 0.05) and genotypes (*P *= 0.06) between cases and controls (Table 1). The data supported a recessive model and individuals homozygous for the *FCGR2A*-131R allele had an increased risk of GCA (OR 2.10, 95% CI 1.12 to 3.77, *P *= 0.02, compared with all others). There was also a trend towards an increase in the *FCGR3A*-158F/V allele (*P *= 0.06) and genotype (*P *= 0.08) frequencies in GCA patients compared to controls (Table 1). The data were consistent with a dominant model and carriage of the *FCGR3A*-158F allele was associated with GCA (OR 3.09, 95% CI 1.10 to 8.64, *P *= 0.03). There were no significant differences in *FCGR3B *or *FCGR2B *allele or genotype frequencies between GCA subjects and controls.

### Linkage disequilibrium at the *FCGR *genetic locus

Significant linkage disequilibrium was observed between *FCGR2A *and *FCGR3A *(*D' *= 0.31, *P *= 0.01) and *FCGR3A *and *FCGR3B *(*D' *= -0.64, *P *= 0.0001) in the control population. The negative *D' *values indicate linkage disequilibrium between the commonest allele of one gene and the least common allele of the second gene (Table 2).

### Association of *FCGR *haplotypes with giant cell arteritis

The distributions of two-locus *FCGR *haplotypes were compared between the GCA and control populations, with a difference approaching statistical significance for *FCGR2A-FCGR3A *(Table 3). Compared with the control frequency of 36%, the *FCGR2A-FCGR3A *131R-158F haplotype was found at increased frequency in GCA patients (50%).

From the haplotype trend regression analysis of *FCGR2A-FCGR3A *haplotypes, the 131R-158F haplotype was found to have a significant effect on the risk of GCA (OR 1.72, 95% CI 1.02 to 2.89, *P *= 0.04), compared to the H-V haplotype as baseline. These are maximum likelihood estimates of the effect of each haplotype assuming a log-additive combined effect of an individual's two haplotypes. Further analysis treating the number of copies of this haplotype as a factor shows that the effect of the haplotype is largely confined to those with two copies (data not shown). *FCGR2A-FCGR3A *haplotypes were analysed under a recessive model, where homozygosity for this haplotype was observed in 12% healthy controls compared with 27% GCA population giving an OR of 2.84, 95% CI 1.33 to 6.06 (*P *= 0.01) when homozygotes were compared with all others.

### Contribution of *FCGR2A *genotype and HLA-DRB1*04 alleles in giant cell arteritis susceptibility

There was evidence of a multiplicative joint effect between homozygosity for *FCGR2A*-131R and HLA-DRB1*04 positivity, consistent with both of these two genetic factors contributing to the risk of disease (OR 2.23, 95% CI 1.09 to 4.58 and OR 2.61, 95% CI 1.30 to 5.22, respectively, from a model including both predictors). Thus, the risk of GCA in HLA-DRB1*04 positive individuals homozygous for the *FCGR2A*-131R allele is increased almost six-fold compared with those with other *FCGR2A *genotypes who are HLA-DRB1*04 negative. There is no evidence of an interaction between these two genetic loci (*P *= 0.19 from a likelihood ratio test).

## Discussion

We have demonstrated a significant association between *FCGR2A*-131RR (OR 2.1, *P *= 0.02) and *FCGR3A*-158F (OR 3.09, *P *= 0.03) with susceptibility to GCA. Haplotype analyses can be more informative in their ability to identify disease predisposing genes. They may also provide additional evidence for the presence of unidentified polymorphic variants that are in linkage disequilibrium with the haplotype and are the true disease-susceptibility variants [36]. Accordingly, homozygosity for the *FCGR2A-FCGR3A *131R-158F haplotype was associated with an almost three-fold increased risk of GCA (OR 2.84, *P *= 0.01).

The FcγRs play important roles in the initiation and propagation of many different immunological and inflammatory processes. The two alloforms (*FCGR2A*-131R and *FCGR3A*-158F) contained within the GCA-susceptibility haplotype encode low affinity variants (FcγRIIa-131R and FcγRIIIa-158F). FcγRIIa is the major phagocytic FcγR in humans and has two co-dominantly expressed alleles, 131H and 131R. The 131H isoform is the only FcγR that can bind IgG2, an antibody subclass that is also a poor activator of the classical complement pathway. *FCGR2A*-131H is, therefore, essential for handling IgG2 immune complexes: individuals homozygous for the 131R-allele have been shown to have an increased susceptibility to various encapsulated bacterial infections, such as *Neisseria meningitides*, *Haemophilus influenzae *and *Streptococcus pneumoniae *[37-39]. FcγRIIIa is expressed on natural killer cells, macrophages, γδ T-cells and a subset of monocytes. Consequently, they may act as susceptibility factors for GCA through a variety of mechanisms, such as an inability to bind IgG2 haplotypes (FcγRIIa-131R) on a background of impaired FcγR-mediated phagocytosis (FcγRIIa-131R and FcγRIIIa-158F) and impaired antigen-dependent cellular cytotoxicity of IgG-opsonised cells (FcγRIIIa-158F). Alternatively, FcγR polymorphisms may modulate endothelial leukocyte ingress, dendritic cell endocytosis and antigen presentation [26], or macrophage/natural killer cell effector functions to IgG containing immune complexes, each of which could separately influence the pathogenesis of GCA.

The same polymorphic variants have also been demonstrated to be associated with susceptibility to a variety of autoimmune diseases. For example, systemic lupus erythematosus is associated with the same *FCGRIIA*-131R, *FCGRIIIA*-158F and *FCGRIIIB*-NA2 alleles, whereas relapses in Wegener's granulomatosis are associated with *FCGRIIA*-131R and *FCGRIIIA*-158F alleles [40]. Flares in both systemic lupus erythematosus and Wegener's may be triggered by infection, particularly chronic nasal carriage of *Staphylococcus aureus *in the latter, but the picture is also complicated by impaired FcγR-mediated clearance of pathogenic autoantibodies and immune complexes.

## Conclusion

Genetic polymorphism within the *FCGR *genetic locus may contribute to the development of GCA. The immunological consequences of these subtle defects in the innate immune system may be enhanced in the presence of immunosenescence, and the increased susceptibility to infection may potentially allow triggering of the inflammatory process in GCA. Additional studies from other ethnic groups will be required to confirm these genetic associations.

## Abbreviations

CI = confidence interval; FcγR = Fc gamma receptor; GCA = giant cell arteritis; NA = neutrophil antigen; OR = odds ratio.

## Competing interests

The authors declare that they have no competing interests.

## Authors' contributions

AWM participated in the design of the study, oversaw all aspects of the laboratory work, analysed the data and prepared the manuscript. JIR, AW and SJB undertook the genotyping assays on DNA prepared in the laboratory of WERO and JHB provided additional statistical support. MAG-G and JM participated in the collection of clinical data and the recruitment of patients into this study. WERO and JDI participated in the design of the study, interpretation of the results and writing of the final manuscript.

## Figures and Tables

**Table 1:** Genotype frequencies (number and proportion) in subjects with giant cell arteritis compared to controls

Gene	Genotype	Control (*n *= 132)	GCA (*n *= 85)	*P *value
*FCGR2A*-131	RR	29 (0.23)	32 (0.39)	
	RH	63 (0.51)	33 (0.40)	0.06
	HH	32 (0.26)	18 (0.22)	
*FCGR3A*-158	FF	47 (0.41)	40 (0.48)	
	FV	49 (0.43)	38 (0.46)	0.08
	VV	19 (0.16)	5 (0.06)	
*FCGR3B*-NA	22	49 (0.45)	36 (0.44)	
	21	50 (0.46)	35 (0.43)	0.77
	11	10 (0.09)	10 (0.12)	
*FCGR2B*-1206	GG	59 (0.49)	36 (0.44)	
	GA	47 (0.39)	34 (0.41)	0.77
	AA	15 (0.12)	12 (0.15)	

GCA, giant cell arteritis.

**Table 2:** Pairwise linkage disequilibrium measures (*D'*) calculated from the control group

	*D*' measures for the control population		
	*FCGR2A*	*FCGR3A*	*FCGR3B*

*FCGR3A*	**-0.31**		
*FCGR3B*	-0.07	**-0.64**	
*FCGR2B*	-0.03	0.05	**-0.37**

Values of 0.3 and higher highlighted in bold.

**Table 3:** Estimated pairwise haplotype frequencies in giant cell arteritis compared with healthy controls

Genes	Haplotype	Control	GCA	*P *value
*FCGR2A-FCGR3A*	131R-158F	0.360	0.502	
	131R-158V	0.135	0.078	0.07
	131H-158F	0.246	0.208	
	131H-158V	0.259	0.212	
*FCGR3A-FCGR3B*	158F-NA2	0.347	0.444	
	158F-NA1	0.272	0.268	0.16
	158V-NA2	0.339	0.225	
	158V-NA1	0.042	0.063	
*FCGR3B-FCGR2B*	NA2-1206G	0.426	0.343	
	NA2-1206A	0.255	0.317	0.30
	NA1-1206G	0.255	0.311	
	NA1-1206A	0.064	0.029	

Pairwise haplotypes produced from four biallelic markers (*FCGR2A*-131H/R, *FCGR3A*-158F/V, *FCGR3B*-NA2/1 and *FCGR2B*-1206G/A) denoted in the order they occur at the *FCGR *locus. Thus, 131R-158F indicates a haplotype containing *FCGR2A*-131R and *FCGR3A*-158F alleles. Empirical *P *values were obtained from a heterogeneity test statistic incorporated in the Permutation and Model-free analysis program after 1,000 permutations.
